# Multi-Segmented Nanowires: A High Tech Bright Future

**DOI:** 10.3390/ma12233908

**Published:** 2019-11-26

**Authors:** Da-Shuang Wang, Aiman Mukhtar, Kai-Ming Wu, Liyuan Gu, Xiaoming Cao

**Affiliations:** The State Key Laboratory of Refractories and Metallurgy, International Research Institute for Steel Technology, Collaborative Innovation Center for Advanced Steels, Wuhan University of Science and Technology, Wuhan 430081, China; waloneds@sina.com (D.-S.W.); ; (L.G.);

**Keywords:** multi-segmented NWs, microstructure, magnetic properties, mechanical properties, electrochemical deposition

## Abstract

In the last couple of decades, there has been a lot of progress in the synthesis methods of nano-structural materials, but still the field has a large number of puzzles to solve. Metal nanowires (NWs) and their alloys represent a sub category of the 1-D nano-materials and there is a large effort to study the microstructural, physical and chemical properties to use them for further industrial applications. Due to technical limitations of single component NWs, the hetero-structured materials gained attention recently. Among them, multi-segmented NWs are more diverse in applications, consisting of two or more segments that can perform multiple function at a time, which confer their unique properties. Recent advancement in characterization techniques has opened up new opportunities for understanding the physical properties of multi-segmented structures of 1-D nanomaterials. Since the multi-segmented NWs needs a reliable response from an external filed, numerous studies have been done on the synthesis of multi-segmented NWs to precisely control the physical properties of multi-segmented NWs. This paper highlights the electrochemical synthesis and physical properties of multi-segmented NWs, with a focus on the mechanical and magnetic properties by explaining the shape, microstructure, and composition of NWs.

## 1. Introduction

One-dimensional (1-D) nanostructures contain an important class of nano-science and technology. Nanowires (NWs) are defined as a 1-D structure with a transverse limit of less than 100 nm [1]. Typical NWs have an aspect ratio of more than 1000, so they are often referred to as 1-D nanomaterials [2]. Depending on the type of material they can be classified as metal, semiconductor, and insulator. NWs are fabricated either by direct synthesis or assembly methods [3,4]. Electrochemically fabricated NWs play a significant role in nanotechnology, and become a vital part of human life.

Since the publication of Kelly et al. [5], the development of composite materials became the topic of interest. Due to the emergence of composite nano-materials in recent years, and due to their interesting structure and numerous physical properties, this has promoted the research of multi-functional macroscopic engineering materials, and made progress in growth of nano-composite materials [6]. Now, it is hardly a surprising fact that the multilayer and sandwich nano-structures have gained considerable attention, both in industrial and scientific communities, owing to their excellent properties. Recently, a lot of researchers have pointed out the significance of multilayers magnetic/non-magnetic nanostructures, and it became a topic of research for theoretical and experimental studies. Due to noteworthy magnetic exchange interaction and giant magnetic resistance phenomena was observed in such nanostructures (i.e., NWs) they have many desirable applications in magnetic barcoding systems, electrical switching devices, magnetic sensors, dense storage medium and giant magneto-resistance (GMR)hard drives.

Multi-segmented NWs can compose of layers of different materials (i.e., polymers, metals, semiconductors and dielectrics), and usually they are a few nanometers diameter in thickness. Precise control of multi-layer segments on nanometer scale makes them promising to enhance mechanical and magnetic properties to a desirable degree. Several researchers named them differently, for example, multi-component NWs, nano-barcodes, nanomotors, and multi-segmented or bamboo nanostructures, but the basic concept stays the same [7]. Numerous synthesis methods were used for the formation of hetero-structured NWs for device applications [8,9,10,11,12,13]. After the synthesis of barcode metallic NWs by Pen et al. [14], electrodeposited multi-segmented NWs were exhaustively investigated [15,16,17,18]. Multi-segmented NWs, usually prepared using the templates assisted method, template synthesis of NWs array contains the electrochemical reduction of one or more ions of desirable metals inside the nano-pore channels of an insulating membrane [17]. NWs prepared from templates spontaneously immobilized in an ordered way because of the well-arranged and ordered structure of the template. Thus, multi-segmented NWs prepared from templates can significantly be used for device fabrication and have a wide range of applications [17,18,19,20,21,22]. Multifunctional NWs display unique responses to the external filed such as alternating current(AC) or Direct current (DC) electric field or magnetic field or lasers in the near-infrared or ultraviolet range [15,23,24]. By changing the composition, shape and microstructure, their response to various fields can be modified as required. Some metal NWs (such as striped metal NWs [14,25], alloys NWs [23], and NWs super-lattice barcodes [26]) can decode their distinct patterns and create corresponding barcodes, which can be used in sensors [27]. Lai et al. [28] showed that the porous Pt-Cu NWs possessed higher stability and catalytic activity than pure Pt NWs.

During recent years the characterizing of the mechanical properties of NWs was an overwhelming task for researchers, because of poor knowledge of the deformation mechanism and the difficulties faced during the control and manipulation of samples during testing. In some recent studies the obtained mechanical tests results contradict the theoretically predicted values. Thus, it became extremely difficult to precisely characterize the mechanical properties of NWs, because of an absence of the key understanding of their underlying and size-dependent behavior. However, due to the increased advancement in the characterization techniques in recent decades, it has facilitated substantial progress towards the understanding of NWs’ mechanical properties, such as elasticity, deformation behavior, strength and plasticity. Therefore, the research on mechanical properties of nano-materials enhanced our knowledge of materials at the nano scale, which was not possible to observe in conventional bulk materials.

To the best of our knowledge, there is no review of the electrochemical synthesis, micro structural behavior and physical properties of multi-segmented NWs. Electrodeposition of multi-segmented NWs was discussed by explaining the dual bath and single bath technique. The deposition carried in single bath was operated by techniques like potentio-static, galvano-static and pulse potential, and by periodically varying the potential or current values the desired structural or compositional layer obtained, while in the dual bath technique the different electrolytes were used for deposition of multilayer structure. This review mostly focuses on the materials, that is, Ni, Fe, Co, CoFe, and NiFe, etc, which were electrodeposited as magnetic segments, whereas Cu, Pt and Ag were electrodeposited as nonmagnetic segments. The microsturucture of the multi-segmented NWs was discussed, and we focused on the mechanical and magnetic properties of multi-layered NWs. This study shows that the research focuses on microstructure and mechanical properties of 1-D nanostructures, and will help further in the use of such materials in magnetic and sensing devices.

## 2. Advanced Fabrication of Nanowires

### 2.1. Template Based Synthesis

#### 2.1.1. Anodic Aluminum Oxide (AAO)

**Figure 1:**
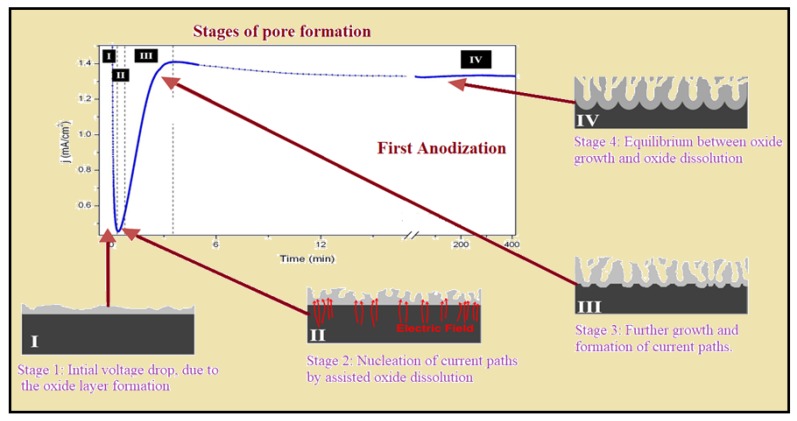
Stages of pore formation on aluminum layer due to oxidation during the first anodization.

From the last few decades, the template-based synthesis of NWs has been gaining attention due to its straightforward technique and cheap synthesis method. Possin et al. [29] were the first to electrodeposit Sn NWs using tacked mica films in 1970; after that, it became a versatile technique used to fabricate 1-D nanostructures such as NWs, nanotubes and nanorods. Since the introduction of honeycomb alumina structures by Masuda et al. [30] in 1994, nano-porous alumina has been widely used as a template for the deposition of various 1-D nanostructures [31,32]. Generally, there are two methods for the fabrication of AAO: (i) Pre-patterned anodization [33] and (ii) two step anodization process [34]. Though the pre patterned anodization could fabricate well-ordered square, hexagonal and triangular pores, it is time consuming and based on an expensive lithography technique [35]. On the contrary, the two step method is cheap and a simple procedure for preparing high quality AAO, as proposed by Masuda and Fukuda [34]. Figure 1 show the stages of pore formation during the first anodization process.

For anodization of alumina the following reactions at the interface take place due to migration of alumina cations (Al^3+^) and oxygen containing anion (O^2−^ or OH^−^).

(a) The reactions that occur at the metal–oxide interface due to Al^3+^ ions migrating outwards towards the metal–oxide interface are as follows [36,37]:

Al → Al^3+^ + 3e^−^ (1)

2Al^3+^ + 3O^2−^ → Al_2_O_3_ (2)

(b) The reactions that occur at the oxide–electrolyte interface due to O^2−^ or OH^−^ migrate inwards towards oxide–electrolyte interface are as follows [37,38]:

2H_2_O + 2e^−^ → 2OH^−^ + H_2_ (3)

O_2_ + 2H_2_O + 4e^−^ → 4OH^−^ (4)

Al^3+^ + 3H_2_O → Al_2_O_3_ + 6H + 6e^−^ (5)

Al + 6OH^−^ → Al_2_O_3_ + 3H_2_O + 3e^−^ (6)

2Al + 3O^2−^ → Al_2_O_3_ + 6e^−^ (7)

The porosity in AAO is defined as [38]

(8) $$P=\frac{S_{pore}}{S_{total}}=\frac{\pi D_{p}^{2}}{D_{int}^{2}2\sqrt{3\;}}$$

where *D_int_* is the interpore distance and *D_P_* is the pore diameter. After the two step anodization the porosity in AAO is about 10%, which can be increased by wet chemical eteching in CuCl_2_ and H_3_PO_4_. For the electrodeposited NWs the term packing density or packing fraction was used instead of porosity. The strong acidic or basic solution was used in order to free the NWs out of the template (for further structural investigations), which usually oxidize the magnetic material. In order to avoid such difficulty, a magntic layer was depsoited to protect the magnetic material from oxidation.

#### 2.1.2. Hard and Mild Anodization in AAO

There are three typical regimes for the formation of self-ordered pores in Al film as mild anodization (MA), that is, for 40 V using C_2_H_2_O_4_ the *D_P_* ~45 nm and *D_int_* ~100 nm, for 25 V using H_2_SO_4_ the *D_P_* ~22 nm and *D_int_* ~60 nm, and for 195 V using H_3_PO_4_ the *D_P_* ~176 nm and *D_int_* ~200 nm to 300 nm and *D_P_* ~40 nm to 60 nm (at 100 to 150 V), and using the H_2_SO_4_ electrolyte solution *D_P_* ~22 nm to 50 nm and *D_int_* ~272 nm to 145 nm (at 27 V to 80 V) [39,40]. Table 1 show the anodization potential and self-ordering regime used during the hard and mild anodization.

The importance of high electric field comes in to notice, aimed to produce highly-ordered pore arrays [43]. Hard anodization (HA) was suggested to expand the regime, which was typically used for MA, that is, using C_2_H_2_O_4_ the *D_int_* ~200 nm to 300 nm and *D_P_* ~40 nm to 60 nm (at 100–150 V), and using the H_2_SO_4_ electrolyte solution *D_P_* ~22 nm to 50 nm and *D_int_* ~272 nm to 145 nm (at 27–80 V) [39,40]. However, HA have various industrial applications, such as in automobile engineering, aluminum cookware, textile machinery, etc.; this is because of high speed oxide growth, of approximately 50–100 μm/h [48,49,50]. However, it has not been favored for the formation of nanostructure materials in the academic research due to difficulties in controlling the aspect ratio of pores of resulting AAO. Additionally, the HA of Al film in H_2_SO_4_ showed poor mechanical properties, resulting in cracks and structural defects [39,51]. Due to the high anodizing voltage applied during the HA, the high electric field results in continuously increasing the thickness of oxide layer at the metal/oxide interface, which also limits the length and diameter of the pores [52]. In order to deal with such a difficulty, Han and Shen [39] changed the electrolyte solution during second anodization, using HA as the first anodization and MA as the second anodization [39], which is called diameter modulated AAO. The diameter modulated AAO template was also a great challenge to fabricate; it was usually made by using MA and HA by a change in solution or by cyclic or pulsed anodization by using the same solution. NWs made by the diameter modulated AAO template should exhibit some special features because scattering of conduction electron along the diameter modulated NWs should be different than the straight NWs, because of the shape effect which can change the boundary condition drastically [53]. Hence, it’s important to form well-ordered AAO films with regular pore arrays and inter pore spacing, which can be handled in different ways and applicable for real life applications.

#### 2.1.3. Track Poly Carbonate Membrane

Track poly carbonate membrane is another technique used to synthesize the 1-D NWs. The polyethylene terephthalate, polyimide and polycarbonate film was used as the basic material for the fabrication of track membranes. IC-100 and U-400 (high energy ion accelerators) are used for the mass production of track membranes, with the ion beam energies in the range of 2.5 to 5 MeV/u [54,55,56]. The irradiation of films with heavy ion beam results in the formation of linear track, for pore formation the tracks are exposed to the wet chemical etching such as surfactant etching or electro-stopping technique [57]. The resultant pore size depends on the etching solution concentration, etching time and on the energy of the ion beams. However, the pore’s distribution in track etch membrane is random, not hexagonally well-ordered like AAO; control over pore size is not easy, and also poly carbonate has lower porosity than AAO. It has some similar characteristics features like cylindrical pore formation [58], with good heat and cold resistance, chemical corrosion resistance, and good electrical performance and light transmittance (light transmittance greater than 90%), which can be used for screening particles and precision filtration [59,60]. Due to good chemical stability they are usually used in ionic devices and bio-sensing applications [57]. Unlike the AAO membrane, the advantage of using polycarbonate membrane is that they can easily be dissolved in solvent like dichloromethane, without oxidizing the already electrodeposited magnetic nanostructure [61].

#### 2.1.4. Step Edge Decoration

Step edge decoration (SED) method is another method to produce the metal and metal oxide NWs, by electrodepsoition at the step edge [62,63]. The electrodeposition usually occurs on low surface free energy layered materials like highly oriented pyrolytic graphite surface (HOPG) or MoS_2_. HOPG is a highly desirable material for SED because the defects present on HOPG acts like linear nanoelectrodes, where the electron transfer to the solution interface redox species [64,65]. First, the nucleation formation occurs on the step edges and they coalesce until they are electrochemically grown in to, usually, polycrystalline NWs. For the compounds like Bi_2_Te_3_ and CdSe, the cycling or stripping electrodeposition was reported to be more significant than the direct deposition technique [66,67]. Another method for the fabrication of metal oxide NWs in SED is electrochemical chemical synthesis, in which first desired materials are electrodeposited, for example, MoO_2_, and reacts with the gas phase species (i.e., H_2_S) at high temperature to form MoS_2_ NWs [68]. In order to reduce the diameter of the NWs, they were etched by electro-oxidation technique [63].

### 2.2. Electro-Spinning

Electro-spinning is a simple, unique and versatile top down technique used for fabrication of ultrathin polymer fiber [69] magnetic NWs [69,70,71,72,73], and core-shell NWs [74]. A simple method to fabricate the NWs by electro-spinning contains a spinneret in which a desired precursor was fed through a thin syringe at a constant rate. An electrode (drum collector) was kept a distance from the spinneret, and with the application of high electric filed the precursor solution become electrostatically charged, solidified and left the spinneret and being collected on the surface of the electrode. For the formation of core shell NWs, a coaxial electro-spinning technique was used, three different precursor solution was fed with three syringes, and similarly, after the application of high electric field, the core shell nanostructure was collected at a drum collector [75].

## 3. Electrodeposition and Structure of Multi-Segmented NWs in AAO Template

**Figure 2:**
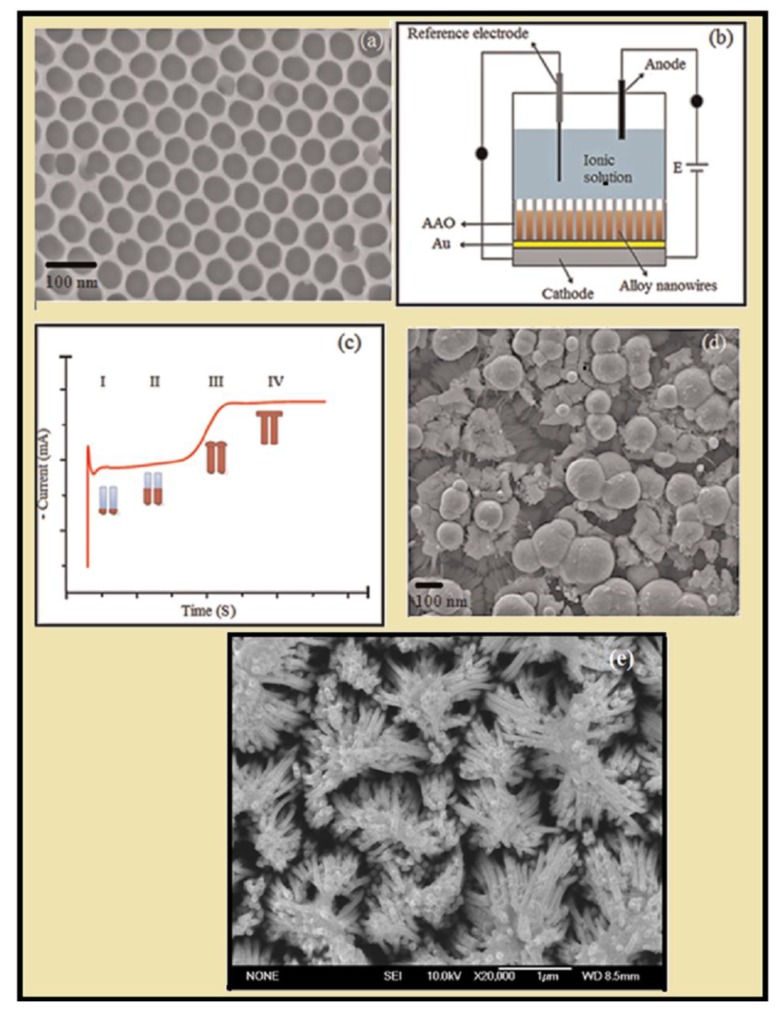
(**a**) A top-view SEM image of PAA template prepared through two step anodization using oxalic acid at 40 V(MA); (**b**) three-electrode cell for deposition of NWs [78]; (**c**) current density vs. time curve observed during the deposition of NWs; (**d**) SEM image of deposited NWs without removal of oxides [78]; (**e**) the SEM image of metal NWs after the removal of oxide in 5 wt% of NaOH solution.

The electrochemical work station was used for the deposition of single/alloy/multi-segmented NWs in AAO template. The three-electrode cell was used for deposition of NWs: (1) AAO template was used as working electrode, (2) graphite/platinum as counter electrode), and (3) Ag/AgCl or saturated calomel reference electrode. Deposition of single or alloy NWs in AAO template is divided into four regions as shown in Figure 2b: Region I, metal ions starts to fill the AAO so the current density abruptly decreases; Region II, as the NWs starts to grow inside the nanopores the current density remain unchanged; Region III, the current density abruptly increases as the metal ions filled up to head; and Region IV, the region where the metal ions deposited on the top of barrier, where the heads reach together and cover the surface area of AAO [76,77]. Figure 2d show the SEM (scanning electron microscopy) image of deposited metal ion without removing the oxide layer.

**Figure 3:**
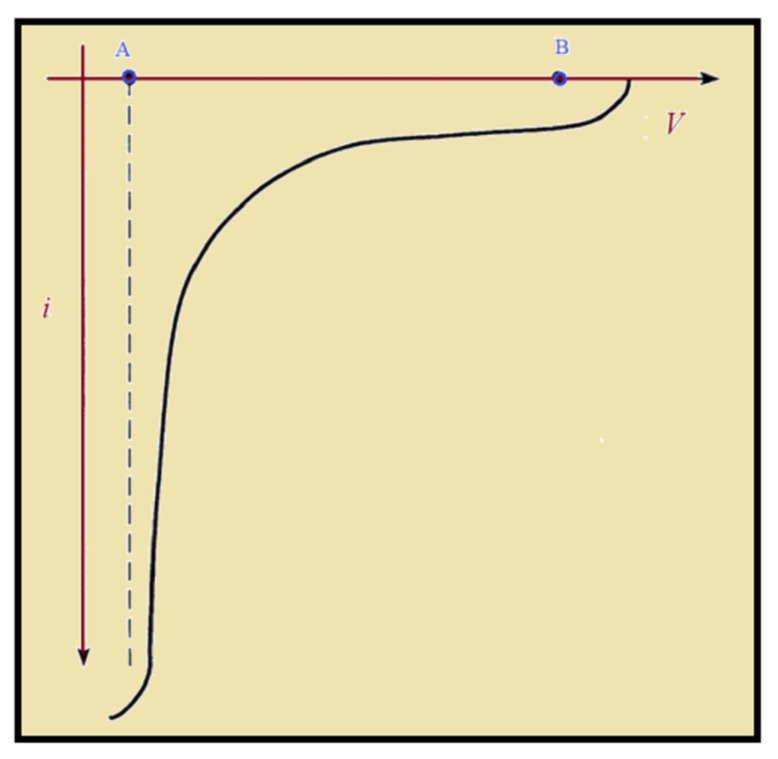
Schematic illustration of *i* (current) vs. *V* (potential) curve for electrolytic solution containing metal A and metal B.

In 1984, the composite structure (Ni-Cu) [79] was actually deposited with the layer thickness in angstroms, and the multilayer structure showed an increase in tensile strength due to the softer Cu metal that was deposited with harder Ni layers. Two years later, two scientists, Yahalom and Zadok [80], pointed out an efficient method to produce the multi-layer structure while using Cu and Ni metal ions. According to the method, a concentrated solution of metal A (assuming that metal is less noble metal than B), was introduced in to the solution of metal B (having very low concentration). Due to the very low concentration of metal B, the rate of reduction of metal B will be slower and only metal B was to be reduced at low polarization potential, and in order to form multi-layer the potential was switched between low and relatively higher potential (Figure 3).

The multilayer structures have attracted much attention because of their unique physical properties. There are different ways to make multilayer NWs, for example, sputtering, chemical vapor deposition and electrochemical deposition [81,82,83]. Among them, electrodeposition is much simpler and less expensive. The formation of magnetic and non-magnetic layers can be achieved without a non-vacuum environment. The quality of multi layers can be increased by adjusting parameters like bath technique, pH of electrolyte, cathode potentials, layer thicknesses and type of substrate [84,85,86,87]. In the multilayer structure single bath and dual bath is used for the deposition.

The electrodeposition rate and oxidation potentials and can be altered by changing the ionic concentrations, according to the Nernst equation [88]:

(9) $$E=E^{o}-2.3\frac{RT}{nF}logQ$$

where *F* is the Faraday constant (96,485 C mol^−1^), *R* is the universal gas constant (8.314 J mol^−1^ K^−1^), *n* is number of mole, and *T* is the temperature.

### 3.1. Dual Bath Deposition

**Figure 4:**
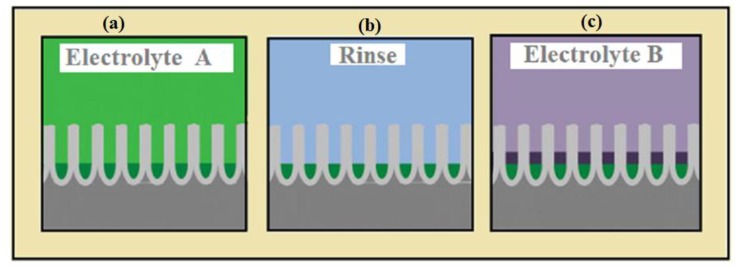
Schematic drawing of multilayer NWs from dual bath deposition, (**a**) the first desired material is electrodeposited from Electrolyte A, (**b**) then rinse the AAO template, (**c**) for the next layer, the separate electrolyte was used to deposit the desired material.

In the case of dual bath deposition, the two different solutions were used for the deposition of multilayers. In the first step, the one desired metal is electrodeposited (Figure 4a); after, this was rinsed in the AAO (Figure 4b); for the next layer, the separate electrolyte was used (Figure 4c); this process was repeated several times as desired multilayers formed [89]. In a dual bath deposition, the oxidation and contamination risk could increase due to unwanted reactions occurring on the substrate during the cleaning and change of substrate. Cleaning is usually done by using deionized water, which could passivate the already electrodeposited layer and make nucleation difficult for the next layer. Additionally, because the displacement reaction occurs and during the deposition of, for instance, an Fe-Cu or Co-Cu multilayer, the more noble metal Cu can displace the already deposited Fe or Co layer [90].

### 3.2. Single Bath Deposition

**Figure 5:**
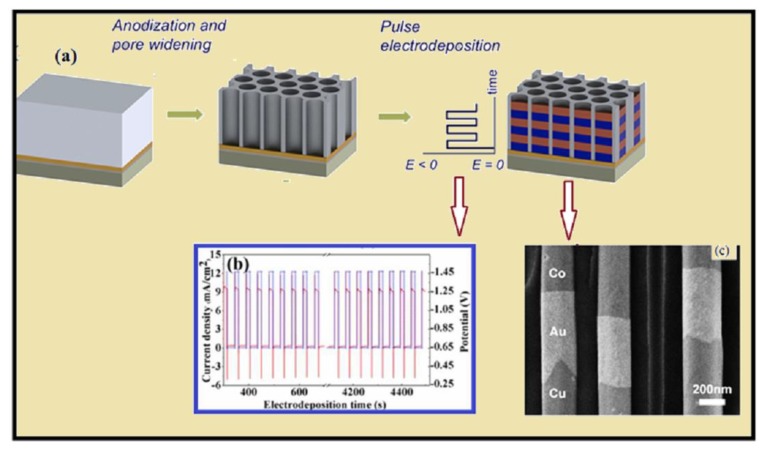
(**a**) The schematic illustration of pulse electrodeposition of multi-segmented NWs in the anodic aluminum oxide (AAO) template. Reprinted with permission from Copyright [102] (2015) American Chemical Society; (**b**) the current vs. time curve for multi-segmented CoCu-Cu NWs. Reproduced from reference [95] by the permission of “Royal Society of Chemistry”; (**c**) SEM image showing Cu/Au/Co segment of NWs, reprinted with permission from [96] Copyright (2014) “American Chemical Society”.

Mostly multi-segments of NWs were deposited by controlling the current or potential, which is known as galvano-static or potento-static mode. While electrodeposition using the potentio-static mode the abrupt voltage change (as pre-set time for required nanometer/micrometer segment) with abrupt current change occurs, this was anodic for some cycles (showing the dissolution of previous electrodeposited segment or hydrogen dissolution). During the galvano-static mode, the abrupt change in current with the potential takes few seconds to reach the equilibrium value in order to consider the example of electrodeposition from electrolytic bath of Co and Cu NWs from the electrolytic solution containing Co^2+^ and Cu^+2^ cations. As Cu is more noble metal than Co, and the redox potential of Co (Co^2+^ + 2e^−^ → Co^0^ is −0.476 V (vs. SCE)) and Cu (Cu^2+^ + 2e^−^ → Cu^o^ is −0.040V (vs. SCE)) is well separated, one can think to deposit pure Co at potential more positive than −0.476 V [91]. Using more negative potential values than −0.476 V, the Cu will deposit along Co. By cycling the potential between high and low pulse, one could form a multilayer with almost pure Cu and Co segments. However, a drawback of this model is that during the deposition of the non-magnetic layer, magnetic layer dissolution occurs [92]. During the deposition of magnetic segments, the nonmagnetic segment (i.e., Cu content) could be minimized by using the rotating cathode which could reduce the agitation. It was shown that during the deposition of Co-Cu layers, excess Cu segment was deposited because the exchange reaction was more than 1.4 nm [93,94]. Figure 5b shows the current density vs. time curve for multi-segmented Co Cu-Cu NWs [95]. Figure 5c shows the SEM image of the Co, Au and Cu interface [96]. Valizadeh et al. [97] has suggested an improved method to calculate the bilayer thickness of magnetic layer (M_L_) and nonmagnetic layer NM_L_ by controlling the charge passed through each layer by using Faraday’s law:

(10) $$m=\frac{MQ}{nF}X$$

where *M* is the molecular weight of M_L_ and NM_L_, *Q* is the corresponding charge to obtain the desired layer thickness, *n* is the number of charge consumed during the deposition process, *F* is Faraday’s constant, and *X* is Faraday’s efficiency. The thickness of metal deposited in terms of current density is

(11) $$\rho =\frac{m}{A\times d}$$

Putting Equation (11) in (10), one could calculate the desired layer thickness of M_L_ and NM_L_ as follows:

(12) $$d=\frac{MQX}{2F\rho A}$$

Cyclic-voltammetry curve was used to obtain the reduction potential of pure M_L_ and NM_L_. Similarly, by using the predetermined values set by Faraday’s law, the combination of galvanostatic/potentiostatic (G/P) mode was used to deposit improved magnetic and nonmagnetic layer [98]. It was seen that by setting the current density of −35.0 mA/cm^2^ for the magnetic layer (i.e., for NiCo alloy) at the G mode, the Cu^2+^ ions does not harm the transport properties of magnetic layer [98]. Further, the P mode was used to deposit the desired NM_L_.

Electrodeposition of layers using single bath was also done by using the alloy electrolyte bath, by periodically varying potential or current. This method of varying current may be applied to many alloy compositions, where the composition of electrolyte is a function of current density. In this technique, the individual segments are proportional to the deposition time, current density, and plating efficiency [99].

Another commonly used method is pulse plating deposition, in which a high voltage (on time pulse) and low voltage pulse (off time pulse) is used to produce the alloy segments (i.e., Co_0.96_Cu_0.04_/Co_0.32_Cu_0.68_ [100]). Like the potentiostatic/galvanostatic deposition, the pulse plating deposition of the nonmagnetic material (i.e., nobler metal) depends on the concentration and potential of the solution. The electrodeposited alloy segments depend on the current density, frequency of the pulse, and the number of on and off time cycles [100,101].

Table 2 shows different current densities and potentials for the deposition of multi-segmented NWs.

### 3.3. Structure of Electrodeposited Multilayer NWs

It is clear from the previous research work that the deposition parameters affects the growth mechanism of metal NWs, and it has technological and fundamental interests. The studies of Tian et al. [112] showed two important points: (1) At low deposition potential Cu, Ag and Au are single crystalline with preferred orientation [110], having an fcc structure, and using high deposition potential polycrystalline structure was obtained; (2) the Ni NWs have a polycrystalline nature and are rather insensitive to deposition potential. They explained that, at lower deposition potential larger critical nuclei are formed, which helped in formation of single crystalline NWs. However, Pan et al. [113] formed single crystalline Ni NWs using high deposition potential, with preferred orientation along [109] direction, they explained that the adsorption of H adatoms on cathode favors [109] growth orientation. Likewise, some other research groups claimed that the electrolyte pH (i.e., from 2 to 6) has no effect on orientation of Ni NWs [114,115]. The formation of fcc Ni, and Ag NWs along [109] was also observed by other researchers [116,117,118]. The growth of single crystalline metal NWs was very well demonstrated by Tan Ming and Xinqi Chen [119]; according to them, the growth of single crystalline NWs occurs on atomically rough planes like hcp [1010] and fcc [110] rather than smooth planes like hcp [0001] and fcc [111]. The reason for this is because the sites for dehydration on atomically rough planes are larger in number than smooth planes [119]. The change in phase transformation of Co NWs was seen (i.e., from hcp to fcc) by changing the deposition parameters [120,121,122], and a group of studies by Mukhtar et al. [123,124,125,126,127] showed that the smaller grain size of Co favors the formation of fcc Co NWs, and larger grain size favors the formation of hcp Co NWs. Similarly, the mixture of hcp and fcc phases was observed in Co NWs, which seems to crystallize in early stage of deposition, and later with the increase in deposition time as the length of Co NWs increases, they crystallize into the hcp phase [128]. Wang et al. [129] studied the expansion behavior of hcp Co NWs arrays along the (1010) plane and fcc Co NWs arrays along the (220) plane. The amount of change in the interplanar spacing of the hcp Co NWs array was found to be larger than the variation in the interplanar spacing of the fcc Co NWs array. The ability to control the thermal expansion of Co NWs is expected to provide new opportunities for the design of magnetic nano-devices.

**Figure 6:**
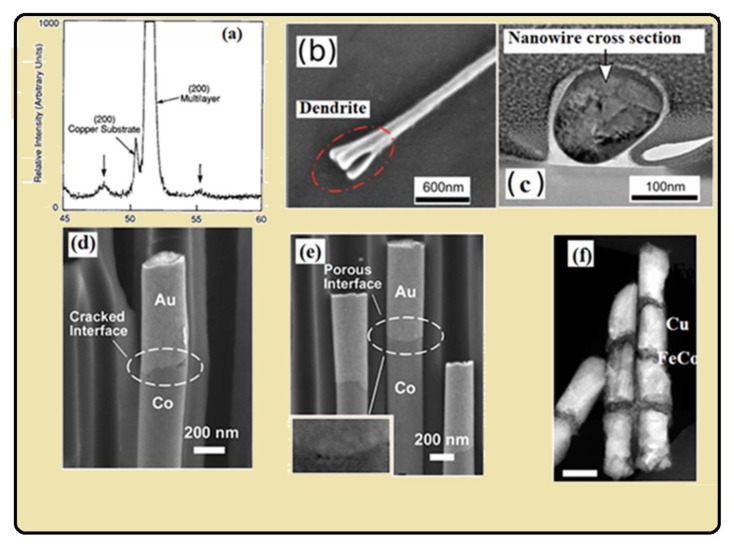
(**a**) X-ray diffraction image of Ni-Cu multilayer composite and two downward arrows with the main central peak show the typical satellites of Ni-Cu multilayer. Reprinted with permission from [130] Copyright “Elsevier”, (**b**) SEM image of NW with dendrite structure, (**c**) Transmission electron microscopy (TEM) image show the cross section of NW. Reproduced from Reference [142] used in accordance with the Creative Commons Attribution (CC BY 4.0) license, (**d**,**e**) SEM of Co-Au NWs shows the defected interface of Co and Au, indicated the porous and cracked interface. Reprinted with the permission from [96] Copyright (2014) “American Chemical Society”, (**f**) Scanning transmission electron microscopy (STEM) image of FeCo-Cu. Reprinted with the permission from [102] Copyright (2015) “American Chemical Society”.

**Figure 7:**
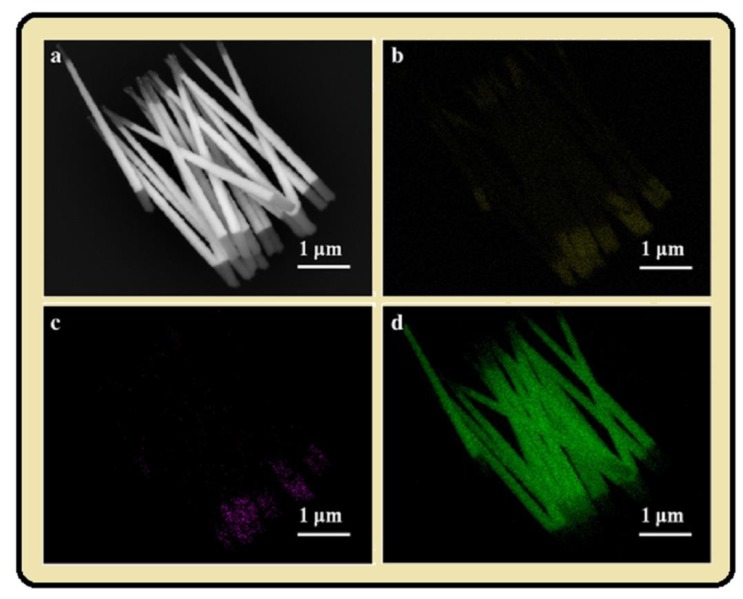
(**a**) SEM image of CoFe/Au/CoFe NWs, EDX mapping of (**b**) Co, (**c**) Fe, and (**d**) Au concentration. Reprinted with permission from [143] Copyright (2013) “Elsevier”.

By having the knowledge of growth of single NWs, as explained in the paragraph above, one can likely understand the growth of multilayer nanostructure. As mentioned in Table 2, the current density, potential and pulse time are the important parameters to form the distinct interface of multilayer NWs, and by controlling these parameters a distinct interface of multilayers could be formed. XRD is a significant tool to study the coherence in multilayers, because if they are coherent there are a lot of them to produce a strong Bragg diffraction peak. Satellites should appear on both sides of Bragg diffraction in the case if the structures are supper-lattice as shown in Figure 6a [130]. The angular shift between the satellites and the Bragg diffraction peak can be explained by $\sin\Delta \theta =\frac{n\lambda }{\Lambda }$, where *λ* is the wave length of X-ray photon, and Δ*θ* is the angular shift, and Λ is the periodicity of superlattice structure. The main peak was seen as intermidiate between the diffraction peaks for Ni and Cu, and satellites can specify that the thickenss of the layers are nearly equal, and they are reproduced by repeating the same experimental conditions cycle by cycle [130]. A lot of work has been done recently on Ni, Co, Fe, Pt and their alloys with Au, Ag, Cu segments [131,132,133,134]. High resolution TEM images allow us to study the cross sectional shape of NWs, which is absoultely cylindrical, but in some studies it deviate from the original shape due to defects in the porous template (Figure 6b,c). Advanced SEM intruments like the Zeiss ULTRA 55 [96] are able to detect the interface defects of multi-segments very clearly, as shown in Figure 6d,e. Both energy-dispersive X-ray spectroscopy (EDS) and energy loss spectroscopy (EELS) gives the chemical composition NWs. The EDS mapping in TEM provides better and higher spatial resolution than the EDS in SEM, and EELS technique even gives more detailed chemical mapping about nanostructure [95,135]. Scanning transmission electron microscopy (STEM) with high angle annual dark field detector depends on material atomic number under study, as the atomic number of magnetic segment (Co) is lower than non-magnetic segment (Au), the magnetic segment appears darker and non-magnetic layer appear as a bright segment (see Figure 2a of [136]). Further analysis of High resolution transmission electron microscopy (HRTEM) showed the polycrystalline nature of Co-Au multilayers, and the growth orientation of Co was interestingly different on [011] and [002] at both sides between Au [210] plane [136]. Figure 7a show the SEM image of CoFe/Au/CoFe NWs, and Figure 7b–d show the EDX mapping of Co, Fe and Au NWs. Similarly, the phase change occurred in NiFe-Au multilayers NWs in NiFe segment from fcc to bcc as the concentration of Fe ions in solution increases [134,137], and for Ni_30_Fe_70_/Cu NWs the increase in Cu thickness from 4 nm to 12 nm and the phase change from bcc (110) to fcc (111) was observed [138]. Meanwhile, depositing the multilayer Co-Cu NWs, the control over deposition parameters, the fcc phase of multilayer NWs was formed [120,139,140,141], which is highly desirable for magnetic applications.

## 4. Properties and Application of Multi-Segmented NWs

### 4.1. Mechanical Properties

**Figure 8:**
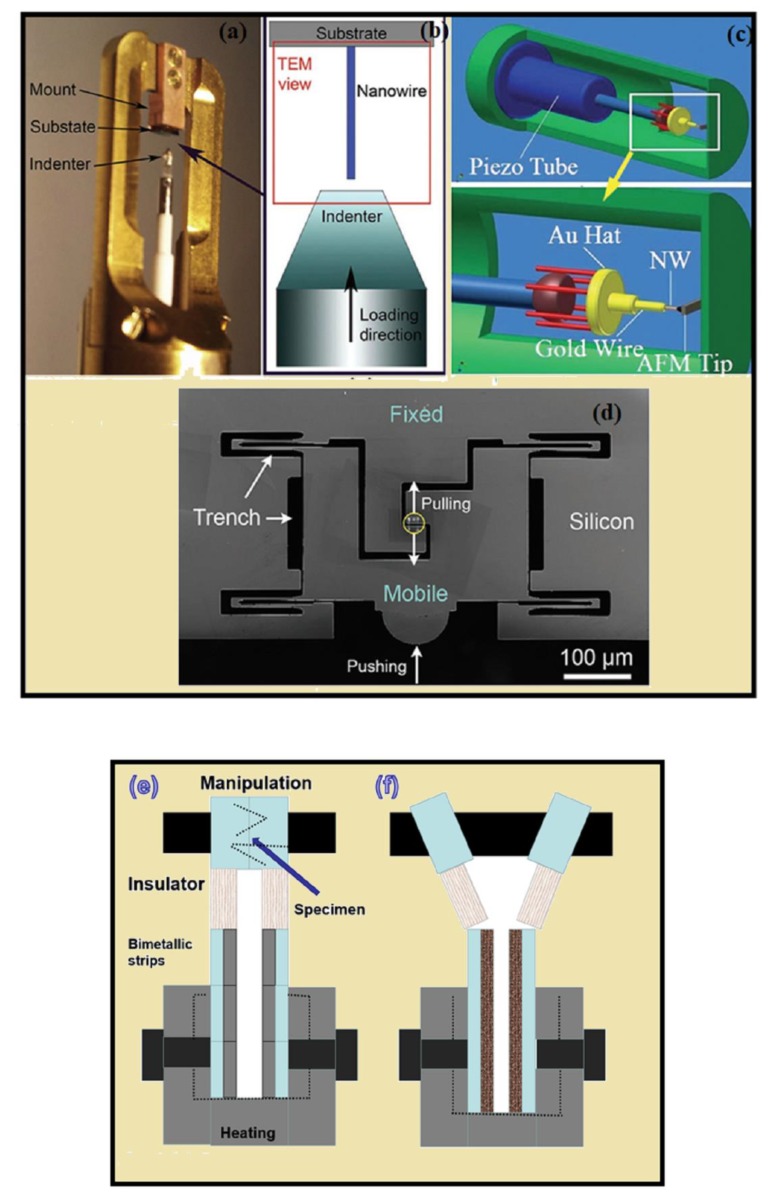
(**a**) The Hysitron PI 95 TEM PicoIndenter and (**b**) the corresponding schematic of a compression test. Reproduced with permission [150]. Copyright (2016) Elsevier (**a**,**c**) A schematic drawing of an in situ AFM/TEM holder used for mechanical tests of NW. Reproduced with permission [148]. Copyright (2012) RSC. (**d**) SEM image of push to pull device, the gray color represent the silicon and black color represents the empty space. Reproduced with permission [150], Copyright (2016) Elsevier. A schematic illustration of the tensile testing mechanism (**e**) illustrates the testing setup before the test and, (**f**) and after the test.

Among the existing techniques, the situ electron microscope (EM) testing is most significant for characterization of NWs because of its high resolution, accurate specimens position, and ability to efficiently monitor the deformation behavior of NW. Along with EM the nanoindentation, atomic force microscopt(AFM), and optical microscopy based techniques are therefore vital methodologies for raising a complete understanding of the mechanical properties of NWs. Figure 8a show image of Hysitron PI 95 TEM PicoIndenter©, and Figure 8b shows schematic illustration, and explains the compression and bending testing mechanism [144,145,146,147]. During the compression testing, a NW attached to a substrate with its axis perpendicular to the surface of the piezo-driven diamond flat punch, a compressive load was applied to the NW by moving the punch towards the NW, which results in buckling of NW. The displacement and applied force of the punch was calculated simultaneously, the elastic modulus can calculate from critical buckling load. Figure 8c shows the schematic of the TEM sample holder, which was fixed with AFM/nano-identaion equipment to calculate the young modulus of NW [148]. A NW was fixed in an Au wire, moved precisely towards the nano-identation tip to make the contact. The nano-identation tip applied the deformation force to the NW. By taking the advantage of the high resolution of TEM, the deformation mechanism of individual NW can directly be observed. Figure 8d shows a schematic illustration of Hysitron PicoIndenter with a push to pull device, which was also used previously [149] in TEM, in order to calculate the tensile strength of submicron wire [150]. The NW was placed between the gap with its both ends fixed between mobile and fixed section of the push to pull device, which was shown by a yellow circle in Figure 8d [150]. As the mobile section moved towards the fixed section by pushing, the gap between the two sections expand, converts the compressive force in to the tensile force [150,151]. The tensile force of NW was also measured by method used by Zhang et al. [152]. The scheme is shown in Figure 8e,f. The individual NW was held between bimetallic strips, made up of two different materials having different thermal expansion coefficients. When the strips were heated (placed in a TEM heating stage holder) they bend in opposite directions and strain is produced, which can be controlled by heating temperature. This method can calculate maximum strain by the images taken by SEM, though this method can’t provide any mechanical data quantitatively [152,153,154]. A lot of current research which focused on the characterization of mechanical properties of NWs did not explain how the environmental factor affects the mechanical test results. Some mechanical results showed that the surface oxidation, temperature, humidity and surface contamination have significant effect on the fracture behavior and elasticity of NWs. Further research on the effect of environmental factors on the mechanical tests will help the current research on mechanical properties of NWs and multi-segmented NWs [150].

**Figure 9:**
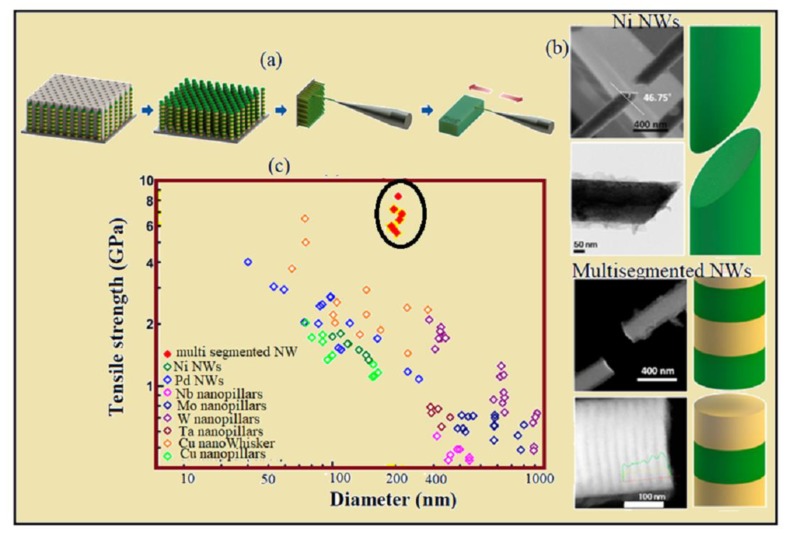
(**a**) schematic illustration of fabrication and preparation of multi-segmented NWs for tensile test (**b**) brittle fracture behaviors Ni and multi-segmented NWs, Ni NW fracture at 30–50° to tensile axis and multi-segmented NW fracture at perpendicular to tensile axis, Reprinted with permission from [168]. Copyright (2016) American Chemical Society, (**c**) Plot of Tensile strength as a function of NW’s diameter, comparing the tensile strength of multi-segmented NWs with [168] Ni NWs [168], Pd NWs [160], Nb nanopillars [169], Mo nanopillars [170], W nanopillars [171], Ta nanopillars [171], Cu nanowhiskers [172], Cu nanopillars [173].

There is an increasing demand for ultra-high strength materials for supporting the nano and micro devices with the fast development of nanotechnology. Reliability and stability of such materials should be certain even under high external stress [117,118,119]. NWs have extraordinary potential for achieving ultra-high strength because of high aspect ratio, comparatively less point defects and dislocations. Previous research with nano-layered composite structures showed an increase in strength as the layer thickness was reduced to some specific value. The reason behind this strengthening could be the lattice mismatch and the young modulus mismatch between the adjacent layers. The young modulus creates a force between a dislocation, and the lattice parameter mismatch introduces mismatch dislocation and stress, which interacts with mobile dislocation. In case single dislocations overcome both barriers, a peak of yield stress occurs [155]. A recent study was conducted to examine the nanoscale deformation behavior of nano-materials [156]. Many attempts were made to study the mechanical strength of single crystalline metal NWs by change in length and diameter of NWs. A well annealed crystal contained 106 dislocations/cm^2^ of the surface, and as the sample approaches this dimension the change in plastic behavior of sample is obvious [157,158]. Reduction in compressive strength by a change in diameter was seen by Rinaldi et al. [159] in single crystalline Ni nano-pillar; similarly, yield strength of single crystalline Pd and Au NWs were increased from 1 to 4 GPa with the decrease in diameter from 180 nm to 40 nm [160]. The effect of change in samples diameter on yield strength (*ρ_ys_*) of metal nano-pillar diameter (*D^−k^*) was described by equation $\rho_{ys}\propto D^{-k}$ [161]. The study of microstructural behavior of NWs was a key parameter to understand the mechanical properties of NWs, and the distribution of twins in single crystalline NWs strongly influences the formation and propagation of dislocations. Wang et al. [150] highlights the relation between density of twins and tensile deformation while studying the fcc metal NWs. Lee et al. [162] and Jang et al. [163] explained the deformation behavior of NWs because of the formation and enlargement of nano-twins using TEM. The deformation behavior of single crystalline NWs was different than polycrystalline NWs, because the grain size of polycrystalline metallic NWs was confined to order of tens of nanometer. The area of grain boundaries increases as decrease in the grain size occurs, which caused the sliding of grain boundaries. Sansoz et al. [164] explained that because of the sliding of the grain boundaries in polycrystalline Ni NWs, they did not show strengthening by change in diameter. The yield strength of polycrystalline material, as if its grain size reduced to a critical value, was determined by inverse Hall–Petch relation [165,166,167]. To deal with the limitation of inverse Hall–Petch relation, multi-layered structure NWs was introduced. The several segments of multilayer NWs produce obstacles in dislocation propagation and grain boundary sliding, which leads to an increase in strengthening of NWs as described in the paragraph above. Recently, An et al. [168] have reported the mechanical properties of electrodeposited multilayered Ni-NiAu NWs. Studies show that by incorporating Au atoms in multilayer NWs makes multi-layered NWs stronger under tensile strength. Mechanical tests showed that for 10 nm segment thick multilayer NWs, the tensile strength reaches 7.4 GPa, which is 10-times higher than metal NWs reported previously [160,169,170,171,172,173] (Figure 9). Thus, it shows that multi-segmented NWs are promising candidates for attaining the ultrahigh strength, although further studies are needed to study the microstructural and deformation behavior of multi-segmented NWs.

### 4.2. *Magnetic* *Properties*

Multilayered NWs have more diverse applications than the single component NWs because the electrical and magnetic properties of the multi-segmented NWs can be controlled by changing the aspect ratio and magnetic interaction between the corresponding layer [91,92]. Moreover the magneto-resistance response of the multi-segments NWs (magnetic and non-magnetic segment), which were usually studied for 2D multilayer structures, can also be attained. Fcc phase of Co NWs are highly desirable in magnetic applications [174], and Co/Cu multilayer NWs showed enhanced coercivity and saturation magnetization ratio instead of single electrodeposited Co NWs [120,139,140,141]. The easy axis of Co-Cu NWs lies perpendicular to NWs’ axis, in contrast from single component Co NWs. While depending on the aspect ratio of Co-Cu NWs, the aspect ratio of Co segment ≥ 1, the easy axis lies parallel to the wire axis and the magneto-static interaction between the segments lies parallel to the wire axis; similarly, when the Co segment is ≤1, the easy axis lies perpendicular to the wire axis and the magneto-static interaction between the segments lies perpendicular to the wire axis [63,94,95,96]. Cho et al. [175] suggests that the magnetic properties of the Co/Cu barcode nanowires can be enhanced with 50 nm pore diameter, which can be attributed to the filed modification of its growth habit. in the case of multi-segment Ni-Cu NWs [176], when they have rod shaped magnetic segments with aspect ratio greater than unity (2.5) they possess large remanence and coercivity. In this case, the segments moments (easy axis) align parallel to the wire axis. For disk shaped Ni-Cu magnetic segments having aspect ratio less than unity (0.9), the magnetic easy axis align perpendicularly to the wire axis. This is possible since dipolar interactions between the magnetic segments (Ni) favors the anti-parallel alignments [176]. Yao et al. investigated the effect of change of diameter on coercivity of Cu-Ni multilayers NWs, deposited by multistep potential technique [177].The Cu part was removed by Fe(NO_3_)_3_ and bare Ni layer was separated on Si substrate in order to find the exact diameter of Ni and Cu layer thickness. This is also a good way to form nano-cylinders of few nanometers in length with low aspect ratios. Magnetization results showed increased in coercivity of NWs [177]. It was also seen that due to inter-wire interactions the multi-segmented NWs corresponds to much lower coercivity than the single NWs [17,91]. The Cu/Ni multilayer NWs [178,179,180,181] arrays, with uniform sub-layer thickness and a distinct inter-layer boundary with obvious vertical magnetic anisotropy was found. When the applied magnetic field is perpendicular and parallel to the NWs, the rectangular ratios of hysteresis loop are 0.701 and 0.101, and the coercivity is 589Oe and 202Oe, respectively [178,179,180,181].

During the last several decades, magnetic materials have been extensively used because of their low costs and large capacity. Magnetic technology has progressed from magnetic tapes to modern hard disks. Magnetic technology has evolved as the size of magnetic particles has been changed from micrometer to nanometer. In analog recording, which is used in magnetic tapes the signals are converted in to magnetic field and the stored information can be changed by changing the magnetic field or other thermal effects. In digital recording, which is used in modern hard drives, the magnetization bit are aligned right/left in parallel recording and up/down in perpendicular recording. The information can be stable up to super-paramagnetic limit of magnetic particles, and to distinguish one bit from another, the magnetic dipole interaction needs to be minimized; in this respect, the magnetic NWs are the ideal candidates in which the aspect ratio can be controlled as required, and the dipolar interaction can be changed as required. Due to the fast development of data storage technology the continuous increase of the memory density was seen during recent decades. The existing memory density of hard drives already approached 1 Tb/inch^2^, [182] so within present 2D models, the theoretical density limit may have almost come. For example, the present limit for magnetic hard drives is 10 Tb/inch^2^ [183], and the further increase in memory capacity might be possible using atypical methods. The utilization of 3D memory devices with the increased number of layers, in which “bits” are stored, might be the potential solution. Magnetic NWs are the smart candidates to realize 3D magnetic memory devices, where the data bits are densely packed in 3D arrays, stored in the form of magnetic domains along their length. Due to the absence of walker breakdown phenomena in planar devices, the operation speed of magnetic NWs are magnitude faster than the planar devices [184,185]. In order to create such NWs, the pinning of domain wall was produced by using diameter modulated NWs, or having the multilayers in NWs in which the data storage densities can be tuned by several folds [186,187].

#### GMR in Nanowires

Multilayer with magnetic (i.e., Co, Ni, Fe) layer [188] are exchange coupled to each other with a number of spacer materials like noble metals (i.e., Au, Cu, Ag) [189], antiferromagnetic metals (Mn, Cr) [190,191,192] and nonmagnetic transition meals (Pd, Ir, Ru, MO, etc.) [191]. Oscillatory phenomena have been seen in almost all examples. The variation in thickness of spacer layer produce dramatic oscillations in magnetic coupling, and the sign of the coupling oscillates as the thickness of non-magnetic layer increases or decreases. The total number of free conduction electron in the magnetic orbital are dependent on the thickness of the spacer layer, as the number of the nonmagnetic atoms increases or decreases, the atomic orbitals in the magnetic layer experience electron deficient or surplus which in turn causes a sinusoidal variation. The degree of exchange interaction varies inversely with the square of the distance among the magnetic layer. In metals, the oscillations produced due to the sharp cut-off in occupancy at the Fermi level. The de Has-van Alphen effect [193] is a special quantum mechanical affect, in which the magnetic susceptibility of a metal crystal oscillates as the intensity of applied magnetic field increases. Oscillations period a geometrical property was determined by cross sectional area of Fermi surface. The simplest form of interlayer exchange coupling is bilinear coupling, which was described in terms of energy. Bilinear coupling, the energy per unit area in the direction of both ferromagnetic layers *L*_1_ and *L*_2_, can be expressed as

(13) $$E_{1,2}=I_{1,2}\frac{M_{1}\cdot M_{2}}{M_{1}M_{2}}\;=I_{1,2\;}\cos\emptyset_{1,2}$$

where *I*_1,2_ is the coupling constant per unit area, *M*_1_ and *M*_2_ is the magnetization of layers *L*_1_ and *L*_2_, and ∅_1,2_ is the angle between both ferromagnetic layers, *I* < 0 favors antiparallel alignment give antiferromagnetic coupling and positive values of I favors parallel alignment and gives ferromagnetic coupling. Another kind of coupling, which favors perpendicular alignment of magnetization *M*_1_ and *M*_2_ of ferromagnetic layers, is called 90° or biquadratic coupling, and can be expressed as

(14) $$E_{1,2}\;=B_{1,2\;}\cos^{2}\emptyset_{1,2},\mathrm{with}\;B_{1,2\;}>0$$

**Figure 10:**
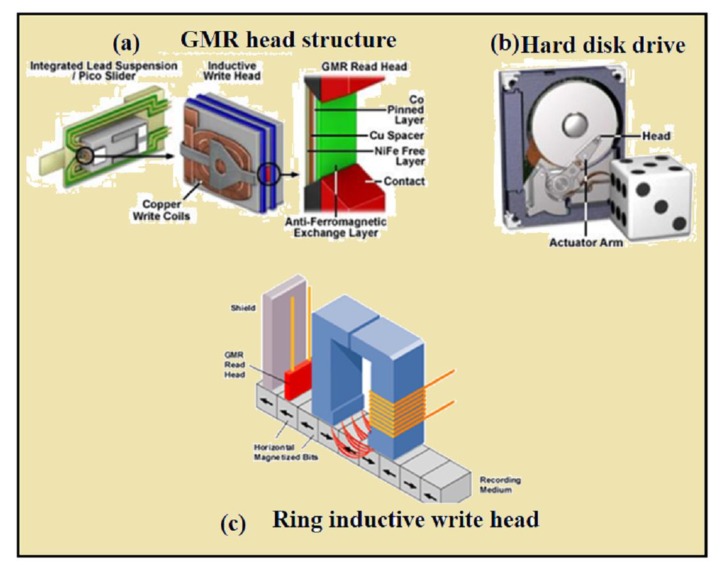
The application of GMR: (**a**) structure of GMR head, (**b**) hard disk drive and (**c**) reading and writing head. Reproduced with the permission from [208] and used in accordance with CC0 1.0 universal (CC0 1.0).

Despite the fact that the interlayer exchange coupling across spacer layer has been known for a long time, in 1986 Grunberg et al. [190] gave clear evidence of antiferromagnetic coupling of Fe layers separated by Cr spacer layer. Later reports of unusual magnetoresistnace effect was observed in layered structures and in 1988, the GMR effect was discovered by two scientists named Peter Gruenberg and Albert Fert [194]. For the discovery of GMR phenomena in 2007, the Nobel Prize was awarded to Peter Grünberg and Albert Fert. GMR is a significant drop in the sample resistance after application of external magnetic field. For example, if the magnetic layers are anti-ferromagnetically coupled (anti parallel) after the application of external field they overcome the coupling aligns the magnetic moments, and change the layers in to parallel alignment, this alignment leads to drop in resistance, called giant magnetosistance. Some of the applications of GMR are shown in Figure 10.

**Figure 11:**
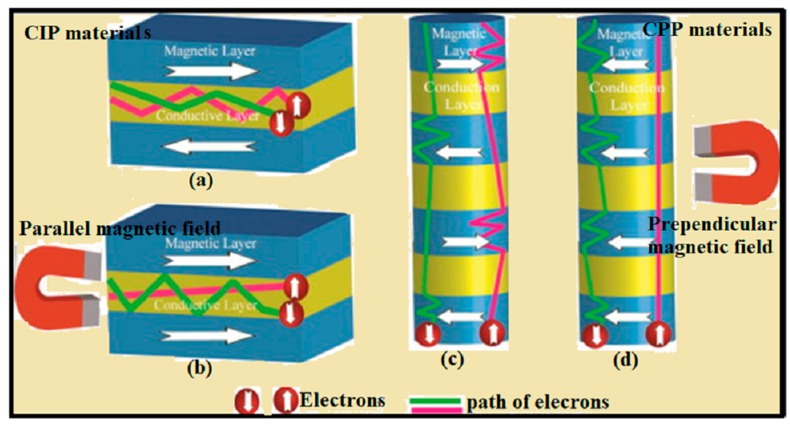
(**a**) CIP GMR multilayer material with no magnetic field applied, (**b**) magnetic field applied parallel to the current (**c**) CPP NW GMR material with no applied magnetic field (**d**) with magnetic field applied perpendicular to the current “Reprinted from [209]. Copyright (2013), with the permission from Elsevier”.

In summary, in GMR materials, spin-down and spin-up electrons experience the equal scattering as they move through the segments of a GMR material. Though, with the application of external magnetic field there is a decrease in probability of scattering of conduction electrons (either spin up or spin down). As there is a decrease in probability of scattering for spin up, there is an increase in scattering probability for spin down electron and vice versa, but the overall scattering probability shows a decrease in resistance to move through multi-segmented material [195]. Figure 11a,b shows the current-in-plane (CIP) and Figure 11c,d shows current-perpendicular to-plane (CPP) GMR structures [196]. For CPP GMR, the magnetic field applied perpendicular to the current flow [196], which changes the spin-flip diffusion length of electrons results in change the total resistance of multilayer material [197]. For CIP GMR, the magnetic field can be applied in the same direction as the current flow, the magnetic field alters the mean free path of electrons, results in change in resistance of multilayer GMR material. CPP GMR materials performance depends on the number alternating multilayers layers, that is, more layers results in higher changes in resistance [198]. Some recent studies show that CPP GMR materials show GMR percentages at low magnetic field than respective CIP GMR materials, but fabrication limitation makes CPP materials (such as multilayer NWs) hard to achieve [199,200].

Due to the structure and high aspect ratio of multi-segmented NWs, they show relatively high GMR ratio with low resistance than traditional multilayers structures [201]. A lot of attempts [80,82,202,203] have been made to enhance the GMR response now it is well known that Co-Cu NWs are promising candidate to boost GMR phenomena [204,205,206]. It was reported that large GMR values was obtain for Co-Cu/Cu multilayers where Cu was deposited at more positive values that its reduction potential although many studies use deposition potential of Cu to obtain GMR results. Chassaing et al. [207] and Toh et al. [98] observed significant GMR at −0.35 V and −0.4 V while depositing Co-Cu/Cu NWs.

**Figure 12:**
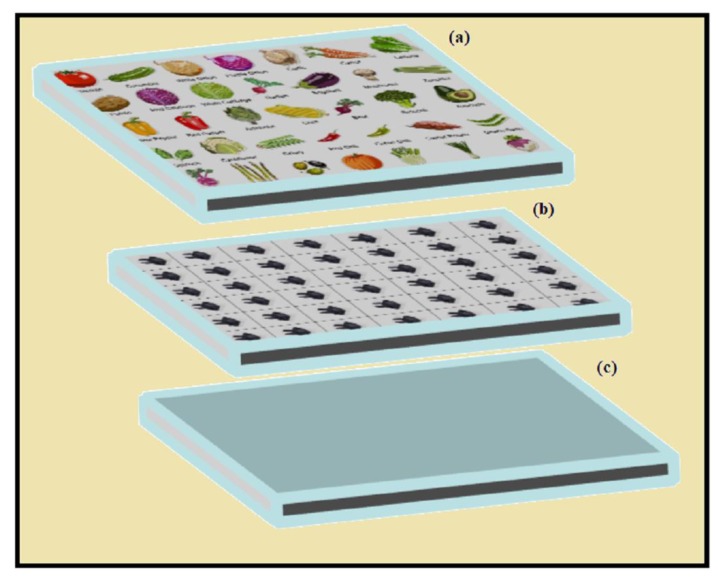
(**a**) Example of GMR sensors: (**a**) nonconductive sheet (**b**) substrate on which sensors are attached (**c**) a ferrous sheet.

Previous studies use magnetic NWs for GMR sensors [98,210,211], Figure 12 is a schematic illustration of GMR sensors [198], in which Figure 12a is a nonconductive sheet on which vegetables are drawn (used in groceries shops etc.), Figure 12b is a substrate on which magnetic sensors are attached and with each sensors a conductor is attached. Whenever a magnetic instrument is placed over image of a vegetable, the sensor placed behind the fruit experience change in resistance, and a ferrous sheet (Figure 12c) which was placed behind the sensor will allow the magnetic instrument to magnetically adhere to the surface of sensor. Moving away or closer the magnetic instrument to the desired sensor (i.e., desired vegetable) will be detected by detected circuit [198]. Recently, Park et al. [212] developed a pressure sensor based on Fe_80_Ga_20_/Cu NW. A GMR sensor was made by depositing Co (1.5 nm) and Cu (2.0 nm) multilayers by e-beam evaporate on silicon grid, its corresponding GMR response was 1.2 mΩ/Oe (calculated sensitivity). When the Fe_80_Ga_20_/Cu NWs were deformed against the GMR sensor, the resistance of the GMR circuit will change as R_3_. A micro-simulation study shows that for the compression of 1 µm and 100 nm the sensing sensitivity of NWs were ~1 mΩ/kPa and ~4 mΩ/kPa [212].

At present, the research of multilayer nanowires in foreign countries is progressing rapidly, and there are still many problems to be solved. For example, (1) the segment thickness is not uniform throughout the whole nanowire length range; (2) usually the cost production of multilayer NWs are too high to bring them practically to the industrial applications. So, in the view of synthesis of multi-segmented NWs, more technology should developed in order to reduce the production cost, and more scientific work should focus in the area to study the microstructure and synthesis of cylindrical nanostructures. (3) It was seen that multilayer NWs have good GMR effect under low saturated magnetic field. However, the saturated magnetic field of NWs at present is generally very high, which seriously restricts its application. (4) Generating GMR also requires low temperatures, which are not conducive to the application of multilayer nanowires, (5) while electrode position, usually a simple electrolytic cell composed of three electrodes, is used. Therefore, for the deep understanding electrodeposition and microstructure, more advanced devices should be used to prepare 1-D nanomaterials. (6) While making nanowires is already possible, putting them together is often very difficult, so we need to determine how to arrange and overlap the NWs, so in future they could apply to specific devices. Surface research should dominate the current trend of NWs research.

## 5. Conclusions

Multi-segmented NWs have attracted attention due to their unique optical properties, high chemical stability, excellent electrical conductivity, good ductility, and great application prospects. The excellent mechanical properties of multi-segmented NWs are adaptable due to the volume expansion, which prevent mechanical degradation and extend cycle life. Additionally, multi-segmented NWs have excellent mechanical flexibility and young’s modulus, which is of great significance for the manufacturing the flexible electronic components. Because of GMR phenomena in multi-segmented NWs, they have great potential in electrical switching devices, magnetic barcoding systems, magnetic sensors, dense storage medium and GMR hard drives.

Due to the characteristics of relatively simple experimental equipment, low costs and easy control of the experimental process, electrochemical deposition research has been carried out extensively and deeply during the last few decades. It has received high attention in the field of preparation of nanomaterials and obtained great development. The versatility of electrodeposition is that it permits to study the variations in orientation dependent properties (i.e., mechanical and magnetic) and could allow fabricating the constant composition as required in multi-segments structures, which is hard to achieve with other techniques like electroless or step edge deposition. The electrochemical deposition is usually operated at low temperatures, and many of the electrolytic baths are required to operate at lower temperatures, so as to diminish the inter-diffusion and ensure deposition of the sharp interface. The most studied multilayered NWs are with Cu segments; however, by carefully understand the different techniques in electrodeposition, a vast range of other multi-segmented alloys would also fabricate in future.

The continuous progress in fabrication of surface and structure properties of AAO will lead to a range of smart AAO based devices in the near future. During the last decade, many secrets of electrochemical processes for the anodization of aluminum have been revealed, but still the mechanism for the formation of self-organized pores of AAO during anodization needs to be well understood and further comprehensive studies are required. Research in this direction is noteworthy because it might provide practical information in designing new rules for architecting porous structure, which is not currently possible with the present anodization techniques. Combined with surface functionalization schemes and advanced anodization techniques, this would expand the application of porous AAOs in many engineering/medicine areas.

The research on the surface effect of NWs is very significant because they can diminish and enhance the mechanical properties of NWs, and their effect on strength and plasticity needs advance study. Additionally, the knowledge on the size effect helps in minimizing the number of defects within the volume of NWs. However it is extremely challenging to exactly measure the defects with in NWs, most importantly the point defects. Thus, a better knowledge of size effects, surface effects and defects and their impact on mechanical properties of NWs, and especially multi-segmented NWs, will remain a noteworthy focus of future research such as sensors and pressure based sensing devices.

## Figures and Tables

**Table 1:** Anodization potential and self-ordering regime using mild and hard anodization.

Hard Anodization				Mild Anodization			
Electrolyte	Anodization Potential	Self-Ordering Regime	Ref	Electrolyte	Anodization Potential	Self-Ordering Regime	Ref.
H_2_SO_4_	40–70	70	[41,42]	H_2_SO_4_	10–25	25	[42,43,44]
H_3_PO_4_	195–235	235	[41]	H_3_PO_4_	160–195	195	[43]
H_2_C_2_O_4_	100–160	120–160	[40,41]	H_2_C_2_O_4_	30–100	40	[44,45,46,47]

**Table 2:** Electrodeposition technique, composition of electrolyte, current densities and potentials for the deposition of multi-segmented NWs.

Multilayer Nanostructure	Composition of Electrolyte (g/L)	Current Density (mA/cm^2^)	Potential (mV)	Technique	Reference
NiCo/Cu	NiSO_4_, CuSO_4_, CuCl_2_, H_3_BO_3_, saachrin (C_7_H_5_O_3_NS)	N/A	V_Cu_ = −400 V_NiCo_ = 1100	Potentiostaic deposition	[103]
CoCu/Cu	116.25CoSO_4_, 8CuSO_4_, 15.5H_3_BO_3_, 0.97H_3_NSO_3_	i_co_ = 60 i_cu_ = 40	V_co_ = −1.5 V_cu_ = −1.3	Potentiostaic deposition	[104]
Co-Cu	90Co, 1.1Cu. In the form of Cu sulfate, 40H_3_BO_3_, and 0.2 of triton X-Loo	i_co_ = 24 i_cu_ = 0.7	N/A	Galvanostatic desposition	[105]
CoNi/Ni NWs	116.25NiSO_4_, 38.7NiCl_2_, 18.6H_3_BO_3_, 19.375CoSO_4_	N/A	V_CoNi_ = 1200 V V_Ni_ = 1200 V	Direct current (Dual bath deposition)	[87]
Ag/Co	23.25CoSO_4_, 118.8K_4_P_2_O_7_, 0.0804AgCN	N/A	V_co_ = −1100 V_Ag_ = −600	Potentiostaic deposition	[97]
Ag/Co	42.15CoSO_4_, 7H_2_O, 0.85AgNO_3_, 30.8CH3COON_4_	N/A	V_co_ = 1000 V_Ag_ = 650	Potentiostaic deposition	[106]
Au/Fe Au	94.08C_6_H_8_OH, 44.08FeSO_4_, 16.24KOH, 210.79 and 0.4925Au	i_Au_ = 0.9 to 10 i_FeAu_ = −15 to −20	V_Au_ = −200, −1100 V_Fe_ = −1250	Galvanostatic Desposition	[107]
Au/Ni	155NiSO_4_, 31H_3_BO_3_, 98.5Au	i_Au_ = −0.34 i_Ni_ = 3.4	N/A	Galvanostatic desposition	[108]
CoPt	46.5CoSO_4_, 14.49K_2_PtCl_6_, 30.07H_3_BO_3_	N/A	V_co_ = −1000 V_pt_ = −350	Potentiostaic deposition	[109]
CoPt/Pt NWs	310CoSO_4_, 20.5[H_2_PtCl_6_]_1/4_, 20–40H_3_BO_3_	N/A	V_copt_ = 1000 V_pt_ = −400	Potentiostaic deposition	[110]
FeGa/Cu NWs	2.28FesO_4_, 7.49 Ga_2_(SO_4_)_3_, 10.29Na_3_C_6_H_5_OH, 2H_2_O(Na_3_-citrate)	N/A	V_FeGa_ = −110 V_Cu_ = −800	Potentiostaic deposition	[111]
Co_0.96_Cu_0.04_/Co_0.32_Cu_0.68_	116CoSO_4_·7H_2_O, 6CuSO_4_·5H_2_O and 45	N/A	15/15 V	Ac Pulse potential	[100]
Ni-Co/Cu	2.5CuSO_4_, 15.45H_3_BO_3_, 112.96NiSO_4_ and 5.73CoSO_4_	i_NiCo_ = −35.0	V_cu_ = 0.585	G/P mode	[98]
